# Effect of modeled reduced gravity conditions on bacterial morphology and physiology

**DOI:** 10.1186/1471-2180-12-4

**Published:** 2012-01-12

**Authors:** Raja Vukanti, Michael A Model, Laura G Leff

**Affiliations:** 1 Department of Biological Sciences, Kent State University, P. O. Box: 5190, Kent, OH 44242, USA; 2 Earth Research Institute, University of California, Santa Barbara, CA 93106, USA

## Abstract

**Background:**

Bacterial phenotypes result from responses to environmental conditions under which these organisms grow; reduced gravity has been demonstrated in many studies as an environmental condition that profoundly influences microorganisms. In this study, we focused on low-shear stress, modeled reduced gravity (MRG) conditions and examined, for *Escherichia coli and Staphlyococcus aureus*, a suite of bacterial responses (including total protein concentrations, biovolume, membrane potential and membrane integrity) in rich and dilute media and at exponential and stationary phases for growth. The parameters selected have not been studied in *E. coli *and *S. aureus *under MRG conditions and provide critical information about bacterial viability and potential for population growth.

**Results:**

With the exception of *S. aureus *in dilute Luria Bertani (LB) broth, specific growth rates (based on optical density) of the bacteria were not significantly different between normal gravity (NG) and MRG conditions. However, significantly higher bacterial yields were observed for both bacteria under MRG than NG, irrespective of the medium with the exception of *E. coli *grown in LB. Also, enumeration of cells after staining with 4',6-diamidino-2-phenylindole showed that significantly higher numbers were achieved under MRG conditions during stationary phase for *E. coli *and *S. aureus *grown in M9 and dilute LB, respectively. In addition, with the exception of smaller *S. aureus *volume under MRG conditions at exponential phase in dilute LB, biovolume and protein concentrations per cell did not significantly differ between MRG and NG treatments. Both *E. coli *and *S. aureus *had higher average membrane potential and integrity under MRG than NG conditions; however, these responses varied with growth medium and growth phase.

**Conclusions:**

Overall, our data provides novel information about *E. coli *and *S. aureus *membrane potential and integrity and suggest that bacteria are physiologically more active and a larger percentage are viable under MRG as compared to NG conditions. In addition, these results demonstrate that bacterial physiological responses to MRG conditions vary with growth medium and growth phase demonstrating that nutrient resources are a modulator of response.

## Background

Bacterial phenotypes result from responses to physical and chemical conditions under which these organisms grow [1-4]. Variation in environmental conditions, for example, changes in temperature [5-7] and availability of nutrients [8-10], alter bacterial responses. Reduced gravity is one such environmental factor that profoundly influences microorganisms [e.g., [11-15]]. Specifically, in this study, we focus on low-shear stress, reduced gravity conditions (< 0.001 Pa; [16]) as a model. This model reflects conditions in which impacts of a cell's microenvironment may be most apparent and is particularly relevant to bacteria in certain parts of the human body (for example, the area between microvilli of respiratory, gastrointestinal and urogenital tracts [17,18]) and those in orbit in spacecraft, such as the International Space Station. The importance of these conditions are multifaceted: serving as an approach for study of sensing of and responses to mechanical stimuli, providing information relevant to human utilization of space (e.g., bacterial growth in spacecraft water systems, implications for human health especially in light of the impacts of space travel on human immune systems), and providing models for conditions microbes experience in parts of the human body [e.g.,[17,18] reviewed by [19]]

To examine biological responses to such conditions, scientists widely rely on ground-based systems, such as rotating wall vessels (RWVs) and clinostats, that create conditions of low-shear, low turbulence and no sedimentation when rotated in a horizontal direction at a specific velocity [20,21]. Conditions achieved through clinorotation are also referred to as weightlessness, modeled reduced gravity (MRG), simulated microgravity, or low-shear modeled microgravity and hereafter are referred to as MRG in this paper. Clinorotation provides a cost-effective, accessible approach to study these conditions relative to space-based research and has been demonstrated to serve as an effective model for examining bacterial responses [19,21].

Previous studies have shown that bacteria grown under either actual reduced gravity or MRG conditions, surprisingly, exhibit resistance to multiple antimicrobial methods [13,22] and become more virulent, which has important potential impacts for human health [23,24], reviewed by [25]. In addition, bacteria under these conditions have enhanced growth [26-28], secondary metabolite production [29], biofilm formation [30] and extracellular polysaccharide production [28]. Other studies have examined changes in transcription (based on microarrays and real time quantitative PCR) and proteomes [e.g., [31-33]] revealing the large scope of responses to these environmental conditions. The mechanisms behind the responses observed are largely unstudied [19]. Lastly, prior research has demonstrated that bacterial responses under actual reduced gravity conditions are similar to those in ground-based studies, demonstrating the effectiveness of this model [26,27].

As noted above, a variety of metrics have been used to evaluate bacterial responses to MRG. However, few of these studies have examined cellular physiological properties or compared responses among different bacterial species (but see [34]; where growth responses of *Sphingobacterium thalpophilium *[a motile strain] and *Ralstonia pickettii *[a non-motile strain] under MRG and NG conditions were compared). Therefore, in this study we examined bacterial physiological properties under environmental conditions created by clinorotation. Specifically, *Escherichia coli *and *Staphylococcus aureus *responses to MRG and normal gravity (NG) conditions under different growth (nutrient-rich and -poor) conditions were examined by analysis of a suite of cellular parameters, including protein concentrations, cell volume, membrane potential, and membrane integrity. Parameters chosen vary with availability of nutrients [9,10,35,36] and are correlated with the physiological status of the cell, including its viability [37-39]. Most of these parameters have not been studied in *E. coli *and *S. aureus *under MRG conditions and they provide critical information about bacterial "health" as well as microenvironmental conditions near bacteria. For example, membrane potential and membrane integrity play important roles in bacterial physiology (such as ATP synthesis, nutrient transport and regulation of intracellular pH), and are essential for viability [40,41].

Bacteria (*E. coli *and *S. aureus*) chosen for this study differ significantly in their physiology and ecology as well as in their cell wall composition, motility, and morphology. Perhaps most importantly, these bacteria differ in the way they respond to changes in concentrations of chemicals (especially nutrients; [42-44]). In addition, *E. coli *(given its motility) has the ability to disturb the quiescent fluid environment that is achieved under MRG conditions while *S. aureus *(non-motile) cannot. Taken together, these experiments provide data at the cellular level that helps us mechanistically understand bacterial responses to MRG conditions.

## Results

**Figure 1:**
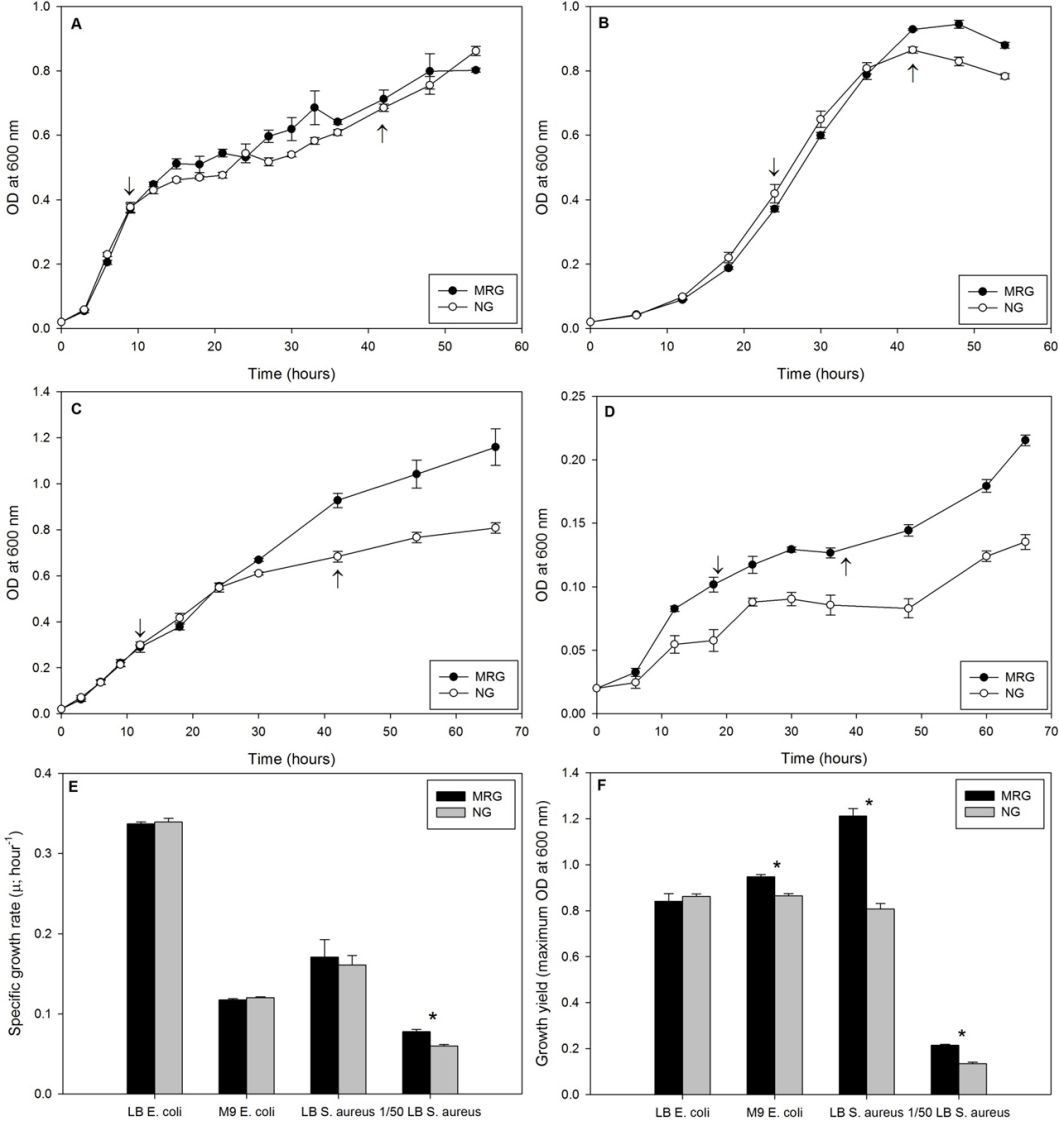
**Bacterial growth curves (based on OD at 600 nm) under modeled reduced gravity (MRG) and normal gravity (NG) conditions, for *E. coli *in LB (**A**) and in M9 minimal media (**B**); for *S. aureus *in LB (**C**) and in dilute (1/50) LB (**D**)**. Down and up-arrows on growth curves indicate the time points at which exponential and stationary phase samples were collected, respectively. Bacterial specific growth rates (μ_max_; h^-1^) (**E**) and growth yields (maximum OD at 600 nm) (**F**) under MRG and NG conditions in various culture media. Values are means (n = 3) and the error bars represent ± standard error of the mean. * = Statistically significant difference between MRG and NG (Student's *t*-test, *P *< 0.05).

**Figure 2:**
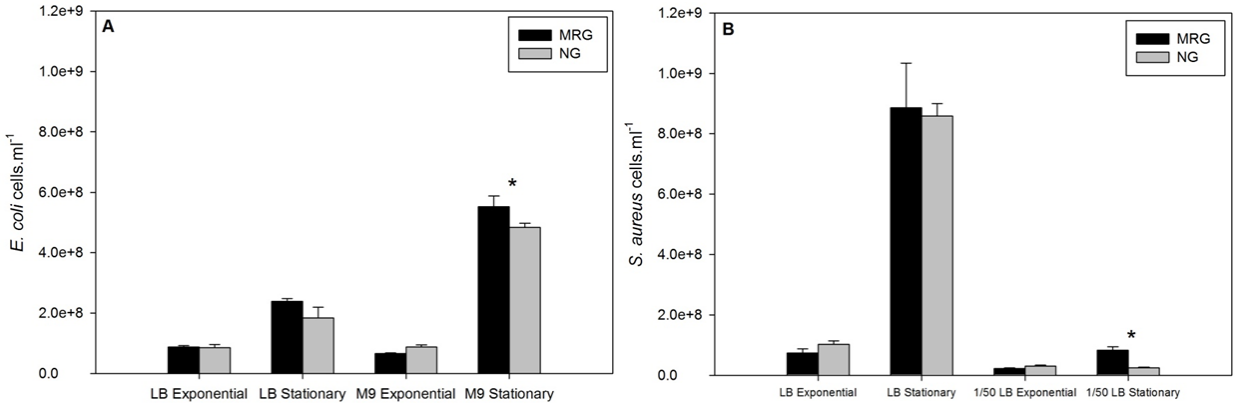
**Abundance of *E. coli *(**A**) and *S. aureus *(**B**) under modeled reduced gravity (MRG) and normal gravity (NG) conditions at different growth phases in different media based on DAPI staining followed by epifluorescent microscopy**. Values are means (n = 3) and the error bars represent ± standard error of the mean. * = Statistically significant difference between MRG and NG (Student's *t*-test, *P *< 0.05).

*E. coli *growth curves (based on optical density [OD] at 600 nm) were similar in Luria Bertani (LB) broth and M9 minimal (M9) media under MRG and NG conditions (Figure 1A and 1B). Although *S. aureus *growth curves were similar under MRG and NG conditions, in diluted LB, OD values were consistently higher, beginning with the exponential phase of growth, under MRG than NG conditions (Figure 1C and 1D). Bacterial growth parameters such as lag duration, specific growth rate, and final cell yield were determined using OD data. Lag duration for both *E. coli *and *S. aureus *grown in either LB or M9/dilute-LB was not affected by MRG condition (as compared to NG control condition) (Figure 1A-D) suggesting that conditions of MRG neither stimulated nor suppressed the duration of the lag phase. Specific growth rate was higher only for *S. aureus *grown in dilute LB under MRG than NG conditions (Figure 1E). Significantly higher bacterial yields were observed for both bacterial strains under MRG than NG, irrespective of the medium with the exception of *E. coli *grown in LB (Figure 1F). Significantly higher numbers of cells (based on 4',6-diamidino-2-phenylindole, DAPI, staining) were achieved under MRG conditions during stationary phase for *E. coli *and *S. aureus *grown in M9 and dilute LB, respectively (Figure 2).

**Figure 3:**
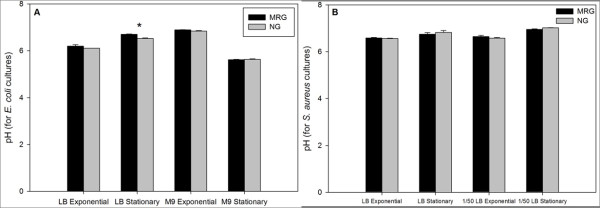
**pH values of *E. coli *(**A**) and *S. aureus *(**B**) culture media under modeled reduced gravity (MRG) and normal gravity (NG) conditions at different growth phases in different growth media**. Values are means (n = 3) and the error bars represent ± standard error of the mean. * = Statistically significant difference between MRG and NG (Student's *t*-test, *P *< 0.05).

Statistically, pH of the *E. coli *and *S. aureus *cultures under MRG and NG conditions were not different in any growth medium with the exception of *E. coli *at stationary phase in LB (Figure 3). In this case, pH under MRG conditions was significantly higher than the pH in NG controls.

**Figure 4:**
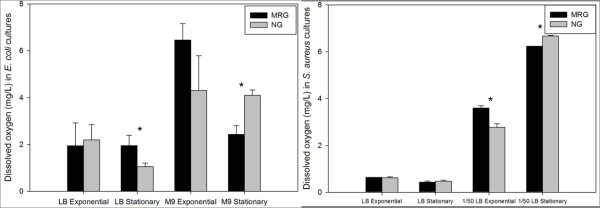
**Dissolved oxygen (DO) levels of *E. coli *(**A**) and *S. aureus *(**B**) culture media under modeled reduced gravity (MRG) and normal gravity (NG) conditions at different growth phases in different growth media**. Values are means (n = 3) and the error bars represent ± standard error of the mean. * = Statistically significant difference between MRG and NG (Student's *t*-test, *P *< 0.05).

For *E. coli *cultures, under MRG compared to NG conditions, dissolved oxygen (DO) concentrations were significantly higher in LB and lower in M9 media at stationary phase, but there were no significant differences in DO at exponential phase in either medium (Figure 4). For *S. aureus *cultures in dilute LB, under MRG compared to NG conditions, statistically higher and lower DO concentrations were found at exponential and stationary phase, respectively, and in LB DO between MRG and NG treatments were not significantly different.

**Figure 5:**
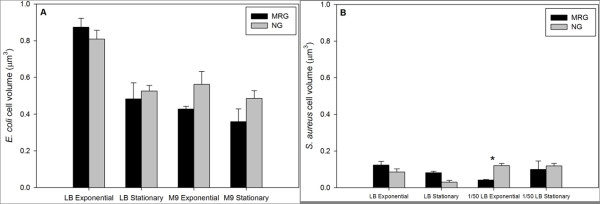
***E. coli *(**A**) and *S. aureus *(**B**) biovolume under modeled reduced gravity (MRG) and normal gravity (NG) conditions at different growth phases in different growth media**. Values are means (n = 3) and the error bars represent ± standard error of the mean. * = Statistically significant difference between MRG and NG (Student's *t*-test, *P *< 0.05).

**Figure 6:**
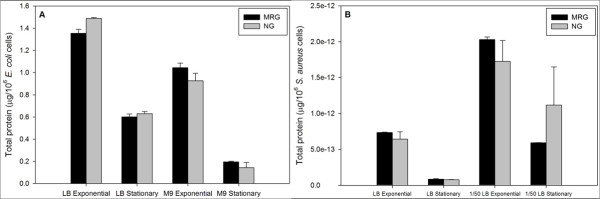
***E. coli *(**A**) and *S. aureus *(**B**) total protein contents under modeled reduced gravity (MRG) and normal gravity (NG) conditions at different growth phases in different growth media**. Values are means (n = 3) and the error bars represent ± standard error of the mean. * = Statistically significant difference between MRG and NG (Student's *t*-test, *P *< 0.05).

There were no significant differences in *E. coli *biovolume (based on DAPI staining and subsequent Metamorph image analysis; Figure 5A) and protein amounts per cell (Figure 6A) when cells were grown under MRG compared to NG conditions at either growth phase or in either medium. On the other hand, *S. aureus *had, on average, a smaller biovolume at exponential phase in dilute LB under MRG compared to NG conditions; there were no other significant differences (Figure 5B). The amount of protein per cell did not differ between MRG and NG conditions for *S. aureus *(Figure 6)

**Figure 7:**
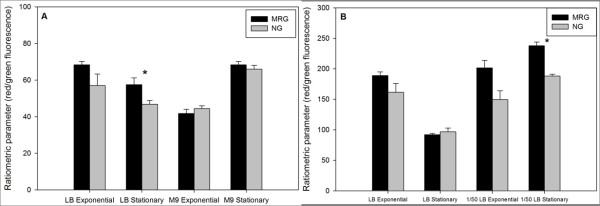
***E. coli *(**A**) and *S. aureus *(**B**) membrane potential (as determined by DiOC_2_(3) staining followed by flow cytometry) under modeled reduced gravity (MRG) and normal gravity (NG) conditions at different growth phases in different growth media**. Values are means (n = 3) and the error bars represent ± standard error of the mean. * = Statistically significant difference between MRG and NG (Student's *t*-test, *P *< 0.05).

Ratiometric membrane potential (MP) measurements (as determined by DiOC_2 _[3] staining followed by flow cytometry analysis) showed *E. coli *and *S. aureus *had significantly higher average MP values at stationary phase in LB and dilute LB, respectively, under MRG as compared to NG conditions (Figure 7). During other growth phases and media conditions, there were no significant differences in MP between MRG and NG conditions for either bacterial species.

**Figure 8:**
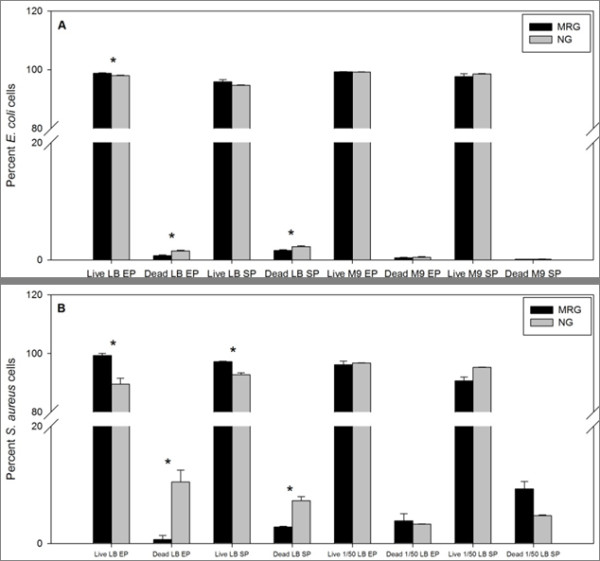
***E. coli *(**A**) and *S. aureus *(**B**) membrane integrity (as determined by SYTO 9 and PI staining followed by flow cytometry) under modeled reduced gravity (MRG) and normal gravity (NG) conditions at different growth phases in different growth media**. Values are means (n = 3) and the error bars represent ± standard error of the mean. * = Statistically significant difference between MRG and NG (Student's *t*-test, *P *< 0.05).

*E. coli *and *S. aureus *membrane integrity **(**MI) measurements (as determined by simultaneous staining with SYTO 9 and propidium iodine) demonstrated that there were more cells with intact membranes under MRG conditions than under NG conditions (Figure 8). However, this significant increase in MI was observed only when bacteria were grown in LB and there were no statistically significant differences in MI in lower nutrient media (M9 and diluted LB). There were strikingly, significantly higher percentages of dead cells of both species during stationary phase in rich medium under NG conditions compared to MRG conditions.

## Discussion

In this study, *E. coli *(motile) and *S. aureus *(non-motile) growth, morphology (biovolume) and total protein expression were examined. In addition, membrane properties, namely membrane potential (MP) and membrane integrity (MI), under MRG conditions were assessed at the single cell-level via flow cytometry. Analyses of basic bacterial functions, such as MP and MI, are critical in understanding bacterial physiological status and viability and previously these properties have not been examined in tandem across bacterial species under MRG conditions. These novel observations provide insight into previously unknown mechanisms that underlie the array of bacterial responses to reduced gravity [reviewed by [19]].

In spite of the diverse suite of attributes that differ between *E. coli *and *S. aureus*, responses of the two organisms were generally similar. Although there are few reports comparing responses of different bacterial species to MRG conditions [45,46], differences in size, physiology, and motility were predicted to impact responses. This tendency to exhibit similar responses suggests that the phenomena observed here represent fundamental ways that bacteria respond to these conditions. Consistency in findings across studies in basic responses (for example, higher cell numbers under MRG) are supportive of this idea [e.g., [26,27,47,48]], but additional comparative studies are needed to determine if these trends hold.

Our observation of higher bacterial numbers (at stationary phase) under MRG conditions is in agreement with observations made by other researchers [e.g. [26,27,47,48]] and suggests that under MRG conditions, lack of sedimentation results in uniform cell distribution throughout the vessel and bacteria having higher accessibility to nutrients thus leads to higher final densities. Differences in bacterial numbers observed in our study depended on the growth medium and growth phase; significant differences between MRG and NG were observed under nutrient poor conditions.

Bacteria respond to nutrient limitation by reducing biovolume (i.e., by undergoing reductive cell division that increases surface-to-volume ratio) [9,49] and protein synthesis [10]. However, no significant differences in bacterial biovolume (except for smaller average *S. aureus *volumes under MRG at exponential phase in dilute LB) and protein amounts per cell were found under MRG conditions when compared to NG conditions. These findings suggest that nutrient limitation, caused by depletion of nutrients in microenvironments around the cells under MRG, was not a significant factor influencing responses.

Membrane potential (MP) is required for a variety of cellular processes, such as ATP synthesis [50], nutrient transport [51], and chemotaxis [52]. In addition, MP is required for survival under stressful conditions, such as exposure to low pH [53] or antibiotics [54,55]. Accordingly, MP is one of the best studied physiological functions in bacteria under a variety of stressful environmental conditions [56-58]. In our study, higher MP values were found under MRG conditions for *E. coli *and *S. aureus *in LB and dilute LB, respectively, and this response was limited to stationary phase. However, *E. coli *grown in M9 minimal media and *S. aureus *grown in LB did not differ in their MP between MRG and NG conditions. This observation is consistent with expectations since MP varies with availability of nutrients [36,57]. We found higher average MP under MRG conditions suggesting that bacterial membranes were more energized under these conditions and which may be due to even distribution of cells that results in higher accessibility of nutrients. Another possibility is that, under MRG conditions, bacteria may be subjected to controlled addition of nutrients (a process similar to fed-batch culturing that is used to achieve high-level of cell densities in fermentation-industry [59,60]) whereby nutrients enter in a more continuous fashion into the microenvironments around cells from the bulk fluid. Regardless of the mechanism, higher bacterial MP under MRG conditions may contribute towards increased survival under the conditions examined.

Another important cellular property examined in this study is membrane integrity (MI). Like MP, higher MI is strongly correlated with bacterial viability [61]. Higher MI was found under MRG conditions for both *E. coli *and *S. aureus *grown in LB, but not in M9 minimal media and diluted LB, respectively. Dramatically higher percentages of dead cells were found under normal gravity conditions in rich media.

Interestingly, in congruence with earlier *E. coli *gene expression studies [33], MP and MI observations are consistent with the observation that *E. coli *grown under MRG conditions exhibits enhanced ability to survive sub-lethal doses of antimicrobial agents [13,22]. As these stress- survival assays require growth of *E. coli *in culture, it is possible that differences in MP and MI account for bacterial phenotypes observed under MRG conditions.

## Conclusions

Documented responses to MRG or microgravity conditions include large scale changes in gene expression as well as more basic responses, such as higher cell numbers. Our study demonstrates that such changes are accompanied by increased membrane potential and lower percentages of dead cells both of which are critical to bacterial population growth. The two species examined, generally, exhibited similar responses. However, responses observed varied with growth phase and were medium-dependent revealing that nutrient availability is a modulator of responses to these conditions. Overall, our data provides novel information about *E. coli *and *S. aureus *MP and MI under MRG conditions and suggest that bacteria are physiologically more active and a larger percentage are viable under MRG as compared to NG conditions. Future studies are needed to elucidate the mechanism leading to increased MP and MI and to determine if these differences are consistently observed regardless of bacterial species and growth conditions. Finally, our findings have implications for fundamental biological responses, namely the ability for living cells to detect and respond to mechanical stimuli [19]. Further study is needed to examine the inter-play between responses to mechanical conditions and other aspects of the environment and to explore potential mechanisms by which such conditions are sensed or detected to determine if they are conserved across taxa.

## Methods

### Bacterial strains

*Escherichia coli *K-12 MG1655 (ATCC 700926), *Staphylococcus aureus *(ATCC 25923)

### Growth media

Full strength Luria broth (LB) and M9 Minimal media (+ 0.4% glucose and 1 μg/ml thiamine) were used to cultivate *E. coli*. Full strength LB and diluted LB (1:50) were used to cultivate *S. aureus*. In this case, diluted LB was used instead of M9 minimal media because M9 did not support the growth of *S. aureus *(data not shown). Filtered deionized water was used for media preparation and sterile technique was used throughout the study.

### Experimental design

Bacteria were initially grown in flasks (with shaking) until the culture reaches early exponential phase and then were mixed with fresh medium. Diluted cultures (optical density [OD] at 600 nm = 0.02) were then inoculated into slow turning lateral vessels with a central core membrane for oxygenation (STLVs, Synthecon Inc., Houston, TX). Completely filled STLVs were then rotated at 40 rpm in a horizontal axis (i.e., perpendicular to the gravitational vector) using a rotating cell culture system (RCCS), so that cells were not subjected to sedimentation and creating a low-shear, low turbulence environment. For normal gravity (NG) controls, another set of STLVs were rotated at 40 rpm in a vertical axis (i.e., parallel to the gravitational vector) using a second RCCS. Triplicate STLVs were used for each condition and bacterial species; vessels were incubated at room temperature.

### Bacterial growth curves

Bacteria were grown in STLVs simulating either MRG or NG conditions. Growth curves were obtained by measuring OD at 600 nm at regular time intervals. Resulting OD data over time for each replicate-sample was analyzed for specific growth rate (μ_max_, h^-1^) and growth yield (maximum absorbance at 600 nm).

### pH and DO measurements

pH and DO of culture media were measured using VWR SympHony (Model SP90M5;VWR Scientific Products, USA) in accordance with the manufacturer's instructions.

### Sample collection

Based on growth patterns of *E. coli *and *S. aureus *in the different media under MRG and NG conditions, two time points that represent exponential and stationary phase were selected for the morphology and physiology analyses. For *E. coli *grown in LB, 9 and 24 hour-time points were chosen to represent exponential and stationary phase, respectively (Figure 1A); and in M9, 24 and 48 hour-time points were chosen to represent exponential and stationary phase, respectively (Figure 1B). For *S. aureus *in full strength LB, 12 and 42 hour-time points were selected as representatives of exponential and stationary phase, respectively (Figure 1C); and in diluted (1:50) LB, 21 and 42 hour-time points were chosen to represent exponential and stationary phase, respectively (Figure 1D).

### Bacterial enumeration

Bacterial number was determined by directly staining with 4',6-diamidino-2-phenylindole (DAPI; Sigma Chemical Co., St. Louis, MO) as described by [62] followed by epifluorescent microscopy.

### Total cellular protein extraction and quantification

Cultures were pelleted by centrifugation. The pellet was washed once with sterile water before it was frozen at -80°C until extraction. Total cellular proteins were extracted by suspending the pellet in 500 μl of 1 × radio-immunoprecipitation assay (RIPA) buffer (Pierce Inc., Rockford, IL) pre-mixed with protease inhibitor, and sonicating the mixture for 18 seconds (three pulses of 6 seconds) using a Microson™ XL2000 ultrasonic cell disruptor (Misonix Inc., Farmingdale, NY). Conditions used for sonication were selected based on a preliminary experimentation and were adequate for obtaining sufficient protein quantities from both bacterial species. After sonication, samples were centrifuged and supernatants were collected. Protein concentration in each sample was measured colorimetrically using a Bio-Rad DC protein assay kit (Bio-Rad Inc., Richmond, CA) with bovine serum albumin as the standard according to the supplier instructions. Normalization was based on cell numbers.

### Measurement of biovolume

Bacteria were fixed by adding one part of sample to the three parts of filter-sterile preservative (that had equal volumes of phosphate buffered saline (PBS) and 8% (w/v) para-formaldehyde) and stored at 4°C. Samples were filtered on to 0.22 μm black polycarbonate filters (Osmonics Inc., Minnetonka, MN) and stained with DAPI as above. Images of DAPI stained cells were obtained using a SPOT RT digital camera (Diagnostic Instruments, Inc., Sterling Heights, MI) attached to an epifluorescent microscope. Cell dimensions (length and width) were measured using Metamorph image analysis software version 4.5r4 (Molecular Devices Co., Downington, PA). Based on the assumption that cells were either spherical or cylindrical with hemispheric ends, biovolume was calculated using the following formula: Volume = (π/4)W^2^(L-W/3) where W is the width and L is the length of a cell [63].

### Ratiometric estimation of membrane potential (MP)

MP was assessed using Bac*Light*™ Bacterial Membrane Potential Kit according to the manufacturer's instructions (Invitrogen Inc., Carlsbad, CA) but with a slight modification. Briefly, bacterial samples were diluted to approximately 10^6 ^cells per ml in filter sterile phosphate buffered saline (PBS). Bacterial suspensions were stained with 3,3'-diethyl oxa-carbocyanine iodide [DiOC_2_(3), final concentration was 30 μM] and incubated at room temperature for 30 minutes. As DiOC_2_(3) in solution contributed to high green background fluorescence, after staining bacterial suspensions, samples were diluted 20 times before they were run on a FACSAria™ flow cytometer (Becton Dickinson Inc., Franklin Lakes, NJ). 488 nm argon laser was used for excitation. Bacteria were identified by forward and side scatter characteristics and gated; gated bacteria were analyzed for their green and red fluorescence signals using FITC (emission collected through 590/30 bandpass) and PE filters/detectors (613/23 bandpass), respectively. Ratiometric parameter was calculated automatically by the FACSAria™ software.

MP was estimated based on ratiometric parameter that is calculated from red and green fluorescence values of DiOC_2_(3). The ratiometric parameter accounts for DiOC_2 _(3) fluorescence dependence on the size of cells (or a clump of cells) [40]. DiOC_2_(3), a lipophilic cationic dye, accumulates in cells and exhibits green fluorescence in the disaggregated state and red fluorescence in the aggregated state [40]. The extent of aggregation (or the amount of red fluorescence) increases with the magnitude of membrane polarization (or ion gradient). Efficiency of MP estimation was verified via the use of a proton ionophore, carbonyl cyanide 3-chlorophenylhydrazone (CCCP, final concentration was 5 μM; [58]).

### Estimation of membrane integrity or permeability

Bacterial samples were diluted to approximately 10^6 ^cells per ml in filter sterile PBS. Diluted bacterial suspensions were stained with SYTO 9 and Propidium Iodide (PI) [64] and incubated for 15 minutes in the dark at room temperature. While SYTO 9 has the ability to penetrate intact bacterial membranes, PI does not. Hence, these dyes can assess bacterial membrane integrity [61]. Samples were analyzed by flow cytometry. Bacteria excited by argon laser (488 nm) were identified on a 2-dimentional dot-plot with forward scatter and side scatter results on y-and x-axis, respectively, and gated. Gated bacterial far red and green fluorescence values were plotted on y- and x-axis of a 2-dimensional dot plot, respectively. Far red and green fluorescence signals were collected using PE-Texas Red and FITC filters/detectors, respectively. Data were subsequently analyzed using FlowJo software (Tree Star Inc., San Carlos, CA).

### Statistical analyses

MRG and NG data for each variable at each time point were compared using student's t-tests conducted in Microsoft Excel and significance was determined if 'P' value is less than 0.05 (n = 3).

## Authors' contributions

RV and LL conceived of and designed the experiments. RV conducted the experiments. MM helped perform the flow cytometry and RV performed the methods for the other data reported. RV and LL analyzed the data. All authors contributed in writing the manuscript and approved its final content.
